# The Validity and Reliability of the Serbian Version of the Smartphone Addiction Scale—Short Version

**DOI:** 10.3390/ijerph19031245

**Published:** 2022-01-22

**Authors:** Aleksandra Nikolic, Bojana Bukurov, Ilija Kocic, Ivan Soldatovic, Sladjana Mihajlovic, Dejan Nesic, Milica Vukovic, Nikola Ladjevic, Sandra Sipetic Grujicic

**Affiliations:** 1 Institute of Epidemiology, Faculty of Medicine, University of Belgrade, Visegradska 26a, 11129 Belgrade, Serbia; nikolicaleksandra89@gmail.com or; 2 Clinic of Otorhinolaryngology and Maxillofacial Surgery, Faculty of Medicine, University Clinical Center of Serbia, University of Belgrade, Pasterova 2, 11000 Belgrade, Serbia; bojanabukurov@gmail.com; 3 Covid Hospital Krusevac, University Clinical Center of Nis, 18000 Nis, Serbia; ilija.k@hotmail.com; 4 Institute of Medical Statistics and Informatics, Faculty of Medicine, University of Belgrade, Dr Subotica 15, 11000 Belgrade, Serbia; soldatovic.ivan@gmail.com or; 5 Hospital for Gynecology and Obstetrics, University Clinical Center “Dr Dragisa Misovic Dedinje”, Heroja Milana Tepica 1, 11000 Belgrade, Serbia; drsladjanamihajlovic@gmail.com; 6 Institute of Medical Physiology, Faculty of Medicine, University of Belgrade, Visegradska 26/II, 11129 Belgrade, Serbia; drdejannesic@yahoo.com or; 7 Institute of Social Medicine, Faculty of Medicine, University of Belgrade, Dr Subotica 15, 11000 Belgrade, Serbia; mv.ciki91@gmail.com; 8 Urology Hospital, University Clinical Centre of Serbia, Resavska 51, 11000 Belgrade, Serbia; nikola.ladjevic@yahoo.com

**Keywords:** smartphone, addictive behavior, medical students, validity, reliability

## Abstract

**Background and Objectives**: Smartphone use has been rapidly increasing worldwide, which has brought possible smartphone addiction into the focus of research. In order to identify potential smartphone addicts, several scales were developed to assess smartphone addiction. Among them, the Smartphone Addiction Scale was frequently used. The study aimed to test the reliability and validity of the Serbian version of the SAS-SV and estimate smartphone addiction prevalence among medical students. **Materials and Methods**: The study was conducted in December 2018 on a convenience sample of 323 third-year medical students. The cross-cultural adaptation was performed following the well-established guidelines for cross-cultural adaptation of self-reported measures. Exploratory factor analysis was used to examine the structure of the questionnaire. Factor extraction was performed by principal component analysis with Varimax rotation. For test–retest reliability, students completed the questionnaire twice within seven days. **Results**: The Serbian version of the SAS-SV showed good internal consistency (Cronbach’s alpha = 0.89) and excellent reliability for test–retest scores (ICC = 0.94, 95% CI = 0.92–0.96). Factor analysis supported the extraction of one factor, which explained 51.538% of the variance. To explore convergent validity furthermore, the SAS-SV was correlated with time indicators of smartphone use. According to cut-off values for the SAS-SV score, 19.5% of students could be regarded as “addicted”, and often spent more time on smartphones and social networks on working days and weekends than “not addicted” students. **Conclusions**: The Serbian version of the SAS-SV is a reliable and valid instrument for detecting smartphone addiction among university students. Further research on this issue is encouraged to enable a better understanding of this ever-increasing public health issue.

## 1. Introduction

There are more than 3 billion smartphone users worldwide, and their number is forecasted to grow by several hundred million in the next few years [1]. Due to their multifunctionality and accessibility, smartphones have become necessary across many life domains and in many professions. Smartphones have become a substitute for computers for some people. For others, they became the most convenient way to entertain themselves anytime and anywhere. Their use has changed the ways of communication and information, and has led to concerns about their excessive use and dependence.

Despite the apparent advantages of using smartphones, a growing literature has found that many people overuse their phones in ways that interfere with their daily activities [2,3,4,5]. The rising use of smartphones and the fact that they provide many features have brought possible smartphone addiction into the focus of research [6].

There are a number of studies on technological addictions, especially on smartphone addiction. However, although the majority of research in this field showed that smartphones are addictive, smartphone addiction has not been mentioned in the Diagnostic and Statistical Manual of Mental Disorders of the American Psychiatric Association (DSM-5) [7] or the International Classification of Diseases (ICD-11) [8] yet. Addiction and substance abuse, in a broader sense, is a complex condition in which there is an uncontrolled use of psychoactive substances despite harmful consequences. The fifth edition of DSM recognizes the category of substance-related and addictive disorders which includes two subcategories, substance-related disorders and non-substance-related disorders. Non-substance related disorders are defined as addictive disorders that do not involve ingestion of a psychoactive substance, commonly referred as behavioral addiction. The only condition currently included in the category of non-substance related disorders is gambling disorder. Gambling behaviors activate reward systems similar to those activated by drugs of abuse and produce some behavioral symptoms that are similar to those produced by the dependence-producing substances [7]. Internet gaming disorder is also included in the DSM-5 as a condition that requires further study. Both gambling and gaming disorders were included in the ICD-11 under the category of disorders due to addictive behaviors [8]. A common set of symptoms associated with behavioral addictions includes salience, mood modification, tolerance, withdrawal, conflict, and relapse [9,10]. There are no official diagnostic criteria for smartphone addiction in the literature, but it is evident that it is related to all three essential aspects of health: physical, social, and mental.

Due to growing concerns about excessive smartphone use, much is being done through research to identify and assess problematic smartphone use, mainly through the development and administration of behavioral assessment scales. A review article by Harris et al. [11] examined 78 existing validated scales that identified or characterized problematic smartphone use or smartphone addiction, and recognized three main groups of scales. The first and the most numerous group included scales that were specifically developed and validated to identify problematic smartphone use or to diagnose individuals with smartphone addiction, overuse, dependency, or attachment (e.g., SAS [6], SPAI [12], PUMP [13], and PMPUQ-R [14]). Many of the scales are similar in their theoretical base, even in items they include. DSM-IV or DSM-5 criteria for substance use or gambling disorder were used to create items in most of these scales to assess addiction. The content domain of most scales is related to dependence-related concepts including craving, tolerance, withdrawal, excessive time spent using, and negative life consequences [11]. The second group consisted of scales assessing smartphone use frequency (e.g., MTUAS [15]). The third group included scales assessing smartphone use motivation and attitudes (e.g., MPAS [16] and MTUAS [15]).

Among scales that are commonly in use, we can highlight the Smartphone Addiction Scale (SAS) [6] and its short version [17] as the most frequently used screening instruments for smartphone addiction. The SAS was developed in South Korea by a group of researchers and clinicians [6]. In order to be more suitable and applicable for research, the SAS was reduced to 10 questions to form the SAS—Short Version (SAS-SV) [17]. The SAS-SV addresses the following areas: daily-life disturbance, withdrawal, cyberspace-oriented relationships, overuse, and tolerance. The advantage of this questionnaire is that unlike other questionnaires, the authors suggested cut-off values to assess smartphone addiction. The SAS-SV is short and easy to complete and has been validated in several languages so far [18,19,20,21,22]. Since there are no empirically validated assessment tools for smartphone addiction available in Serbia, this study aimed to test the reliability and validity of the Serbian version of the SAS-SV, and to estimate smartphone addiction prevalence among medical students.

## 2. Materials and Methods

### 2.1. Translation Process

After obtaining permission from the institutional Ethics Committee (Faculty of Medicine, University of Belgrade) (No. 2650/XII-1) and the author of the original scale, the translation process was conducted following well-established principles [23]. Primarily, the SAS-SV was forward-translated into Serbian by two independent translators (Serbs fluent in English). A group of four experts in a specific medical field discussed and reconciled two translated versions, and a consensus was reached for the first Serbian version of the questionnaire. After that, a fluent English speaker who had not previously seen the original then back-translated the first Serbian version of the questionnaire into English. The two translations obtained were compared to the original English version by a group of experts who discussed the differences and resolved any inconsistencies. Terms and expressions that are common in everyday Serbian language were used. The result was the second Serbian version of the SAS-SV questionnaire (see Supplementary File S1).

### 2.2. Participants and Data

The study was conducted in December 2018 on a convenience sample of students at the Faculty of Medicine, University of Belgrade. Medical students were particularly interesting because smartphone usage in academic and healthcare settings could have a negative impact on student performance and cause potential damage to patient health. The required sample size of 199 was calculated using Epi Info 7 (version 7.2.4.0) (population size: 523 third-year medical students; expected frequency: 29.8% [24]; acceptable margin of error: 5%; design effect: 1). A total of 323 students completed the questionnaire. Of all students that filled out the questionnaire, 77 students were randomly chosen to complete the questionnaire a second time within seven days in order to test the test-retest reliability of the Serbian version of the SAS-SV.

Students were offered the opportunity to voluntarily complete the questionnaire at the beginning of their Epidemiology classes. They were informed about the aims of the study and signed informed consents.

### 2.3. Measures

The Smartphone Addiction Scale—Short Version (SAS-SV) is a self-reported scale with 10 items rated on a 6-point Likert scale (1 = strongly disagree, 2 = disagree, 3 = weakly disagree, 4 = weakly agree, 5 = agree, 6 = strongly agree) [17]. The total score ranges from 10 to 60. The higher scores on the scale indicate a higher level of smartphone addiction. The questionnaire includes 10 questions addressing daily-life disturbance, withdrawal, cyberspace-oriented relationships, overuse, and tolerance. Males are considered addicted with scores higher than 31 (sensitivity of 0.867 and specificity of 0.893), and females with scores higher than 33 (sensitivity of 0.875, and a specificity of 0.886) [17]. The items were selected from the original Smartphone Addiction Scale (SAS), consisting of 33 items [6], based on their validity. The correlation between the SAS-SV and SAS was 0.96 [17].

In addition to this, a specifically developed questionnaire was used to gather sociodemographic characteristics of participants (gender, age, residence, housing, socioeconomic status, grade point average (GPA)) and smartphone usage patterns (see Supplementary File S2). Smartphone usage patterns consisted of questions for which students had to give an estimate of the time they spent using smartphones daily. Questions regarding smartphone usage referred to: (1) time spent using smartphones on a working day, (2) time spent using social networks on smartphones on a working day, (3) time spent using smartphones on weekends, and (4) time spent using social networks on smartphones on weekends.

### 2.4. Statistical Analysis

For the main standard statistical procedures, SPSS 21.0 (SPSS Inc., Chicago, IL, USA) was used, with the difference marked as significant at *p* < 0.05 [25]. The normality of data distribution was assessed visually and supplemented by a Kolmogorov–Smirnov test.

Internal consistency is a measure of the extent to which items in a questionnaire are correlated, and therefore measuring the same concept [26]. To assess interitem correlations, a conventional index of consistency, Cronbach’s alpha, was used. The assessment was also made by excluding one item each time to check the contribution of that particular item to the scale’s homogeneity. A Cronbach’s alpha between 0.70 and 0.95 is considered to be a measure of good internal consistency [26]. Test–retest reliability concerns the degree to which repeated measurements provide similar answers. The intraclass correlation coefficient (ICC) and its 95% confidence interval (95% CI) were used to assess test–retest reliability. Reliability was considered satisfactory if ICC > 0.7 [19]. Exploratory factor analysis was used to examine the questionnaire’s internal structure, since it was the first time exploring the factor solution in a Serbian adaptation. The Kaiser–Meyer–Olkin test and Bartlett’s test of sphericity were computed to determine whether the data were suitable for factor analysis [25]. A factor analysis was used to obtain independent factors, and an item was considered to be loaded on a factor if the matrix coefficient was 0.40 or larger.

Furthermore, for validation purposes, correlations between the total SAS-SV score and time indicators (time spent on mobile phones during working days and weekdays, and time spent on social media on mobile phones during working days and weekdays) were also calculated. The Spearman correlation values (*ρ*) represented were: *ρ* < 0.10, small effect; *ρ* < 0.30, medium effect; and *ρ* = 0.50, large effect [25]. To assess smartphone addiction prevalence among students, we used cut-off values recommended by the original questionnaire’s authors [17] (see above). To compare time spent on smartphones and social networks among “not addicted” and “addicted” students, a χ^2^ test was used.

## 3. Results

### 3.1. Sample Characteristics

Participants were 323 students in the third year of studies at the Faculty of Medicine, University of Belgrade, of which 100 (31.0%) were males and 223 (69.0%) were females. The mean age of participants was 21.0, with a standard deviation of 0.55. Almost half of the students were from Belgrade (49.2%), while the rest were from other regions (Central Serbia, Vojvodina, or other countries). A total of 41.2% of students lived with their parents. Other students (58.8%) lived in their flat, a students’ dorm, or a rented flat/room, or stayed with friends/cousins. Almost two-thirds of students claimed to have a good socioeconomic status (61.3%). The average GPA (grade points range from 6 to 10) was 8.80 (SD 0.72).

### 3.2. Internal Consistency of SAS-SV Questionnaire in Serbian

The results of the item analysis for the Serbian version of the SAS-SV are shown in Table 1. The internal consistency was assessed by Cronbach’s alpha coefficient, which showed an almost excellent level of internal consistency (Cronbach’s alpha = 0.89).

### 3.3. Test–Retest Reliability of the SAS-SV in Serbian

The test–retest reliability of the SAS-SV was examined for 77 students to determine whether the scores derived were relatively stable over time, which was short enough that little real change could be expected. ICC and its 95% CI were calculated as a level of agreement between the initial and seven-day follow-up scores. The test–retest reliability of the SAS-SV questionnaire was excellent (ICC = 0.94, 95% CI = 0.92–0.96, *p* < 0.001).

### 3.4. Exploratory Factor Analysis of the SAS-SV in Serbian

Exploratory factor analysis was used to examine further interitem relationships and dimensions of the questionnaire. Factor extraction was performed by principal component analysis with Varimax rotation (Table 2). The calculated Kaiser–Meyer–Olkin measure of sampling adequacy of 0.89 and the highly significant (*p* < 0.001) Bartlett’s test of sphericity (χ^2^ = 1565.45) indicated that factor analysis was appropriate. All 10 questions showed sufficient loadings on the first principal component to be retained (minimally loading items were question 3 and 8: 0.448 and 0.451, respectively). According to the factor analysis, two factors had eigenvalues greater than 1 (initial eigenvalues 5.154 and 1.085) (Table 3). Together, these two factors explained 62.391% of the total variance (the first factor explained 51.538%, while the other explained 10.853% of the variance).

### 3.5. Convergent Validity of SAS-SV in Serbian

In our sample, the mean SAS-SV score was 23.51 ± 10.22 (minimum 10, maximum 56). The mean time spent on smartphones on working days was 3.83 ± 3.16 h, and 4.53 ± 3.48 h on weekends (Table 4). The mean time spent on social media on working days was 2.69 ± 2.70 h, and 3.29 ± 3.04 h on weekends. The SAS-SV scale significantly correlated with the mean time spent on smartphones on working days (r = 0.31, *p* < 0.001) and weekends (r = 0.32, *p* < 0.001), as well as social media usage on working days (r = 0.39, *p* < 0.001) and weekends (r = 0.42, *p* < 0.001). Associations between time indicators of smartphone use and the SAS-SV total score strengthened the validity of our adaptation.

### 3.6. Smartphone Usage among “Not Addicted” and “Addicted” Students of Medicine

A higher percentage of “addicted” students often spent more than 3 h a day on smartphones and social networks on working days and weekends compared to “not addicted” students (Table 5).

### 3.7. Prevalence of Smartphone Addiction among Students of Medicine

According to the cut-off values for the SAS-SV scores, 63 students (19.5%) could be regarded as “addicted”. Females (22.0%) showed higher percent of potential addiction than males (14.0%), but without reaching statistical significance (*p* = 0.095) (Table 6).

## 4. Discussion

The Smartphone Addiction Scale—Short Version in the Serbian language showed good internal consistency and excellent test–retest reliability in our sample. Reliability measures achieved were excellent (Cronbach alpha 0.89, similar to those obtained by previous studies using the SAS-SV (for the original SAS-SV in South Korea, 0.91 [17]; Turkey, 0.88 [20]; Spain, 0.88 [21]; Belgium, 0.90 (adaptation in French) [21]; and Switzerland, 0.85 (adaptation in German) [27]). The corrected item-total correlations ranged from 0.53 to 0.73, similar to comparable studies using the SAS-SV (0.50 to 0.80 in the original study by Kwon et al., 2013 [17]; 0.43 to 0.76 in the Turkish version [20]; 0.42 to 0.76 in the Arabic version [22]; 0.46 to 0.71 in Spain [21]; and 0.62 to 0.74 in Belgium (French version) [21]. It is possible that these differences were due to the different age groups of participants involved in other validation studies (e.g., high school students [17,20], college students [18,27], students and university staff [21,22], and adults [28]). The results related to test–retest evaluation demonstrated good reliability of the responses (ICC = 0.94), which was in accordance with other studies [18,21]. The final model approach to deciding to extract only one factor in our factor analysis was based not only on a conventional rule to extract all factors with an eigenvalue greater than 1, but we also took into consideration other issues. Mainly, the steepness of the curve on a scree plot (sharp decline) argued against the extraction of the second factor (first 5.154, and second 1.085); for eigenvalues from 0.90 and 1.30, there were other rules that should be considered (e.g., interpretability of the factor solution). We attempted the extraction of a second factor, which led to a split in five questions on each factor, but due to severe cross-loading of all five questions (items 1, 2, 4, 5, and 7 in second factor), we found this factor uninterpretable. Other authors extracted one factor as well, and our results were in accordance with their data [20,22,28].

The participants evaluated in the present study spent about 4 h daily on a smartphone during working days, and even more time on weekends. That represented almost 17% of the day spent using the device, which was significant for this population. Although smartphones could be used as an additional tool for education, many students perceive the smartphone primarily as a leisure device that is most commonly used for social networking, surfing the Internet, watching videos, and playing games. If typically utilized for leisure rather than education, smartphones may be a distraction in academic settings [29]. Excessive smartphone use could be a distraction in clinical settings as well, and could cause negligence and damage to patient health [30], which is important since medical students are future health professionals. Healthcare professionals need to be attentive while performing different procedures on their patients. If they are distracted by smartphones, they could cause errors and harm their patients.

In our study, the variables related to time indicators were significantly and positively correlated with higher SAS-SV scores; however, the correlation was considered of medium strength. Similar results were shown in a Brazilian study [18] and in the study by López Fernández et al. [21], in which all time variables were significantly positively correlated with higher SAS-SV scores in Spain and Belgium. In addition, in our study, “addicted” students spent significantly more time on smartphones than the “not-addicted” ones.

The term “smartphone addiction” was introduced to describe the excessive and psychosocial dysfunctional use of smartphones, which are reminiscent of behavioral addictions. Parallels between the excessive use of smartphones and behavioral addiction are common in studies [6,31,32]. Lin et al. [32] went even further by suggesting diagnostic criteria for smartphone addiction. The smartphone addiction diagnostic criteria consisted of: (1) six symptom criteria (lack of self-control in terms of using, withdrawal, smartphone use for a period longer than intended, and using the phone despite negative consequences), (2) four functional impairment criteria secondary to smartphone use, and (3) exclusion criteria. Findings from the study by Lin et al. [32] indicated that characteristics of smartphone addiction overlapped, to a great extent, with substance-related or behavioral addictive disorders. The unique properties of smartphones, most prominently the access to the Internet and to various applications, contribute to prevalent addictive behavior. Furthermore, Horvath et al. [33] even provided evidence for distinct structural and functional correlates specific to behavioral addictions in individuals who met the psychometric criteria for smartphone addiction.

On the other hand, there were studies that questioned the concept of smartphone addiction [34,35]. Panova and Carbonell [34] stated that the issues associated with the conceptualization and acceptance of technological and behavioral addictions are, to a great extent, related to the terminology. “Smartphone addiction” in comparison to tobacco and heroin addiction is certainly not that severe and with such health consequences. However, there is no other accepted term for such a behavior that manifests as a lack of self-control, attachment, overuse, and harmful consequences. Therefore, due to the lack of a better word “addiction” has become an accepted umbrella term. The authors think that the use of the term “addiction” may misrepresent the severity of the disorder and misguide the research and treatment efforts [34], which leads to overpathologizing normal behaviors [36]. Therefore, Carbonell and Panova [34] suggest the use of the term “problematic smartphone use”.

Even though there is still considerable controversy regarding the term “smartphone addiction”, it is still the most common term in the literature of the field. Accordingly, we decided to use the term “smartphone addiction” in our study to describe excessive smartphone use with a negative impact on daily life functioning. In our study, we opted to use the Smartphone Addiction Scale—Short Version to assess smartphone addiction among medical students.

Regarding the rating scale, we found a high potential prevalence of smartphone addiction (19.5%). The SAS-SV was also used to determine the prevalence of smartphone addiction among students in other countries such as Switzerland (16.9%) [27], China (29.8%) [24], Brazil (33.1%) [18], and Saudi Arabia (71.9%) [37]. López Fernández [21] found a prevalence of excessive smartphone use of 12.5% in Spanish students and university staff and 21.5% in Belgian students and university staff. Among Chinese adults, the prevalence assessed using the same instrument was 38.5% [28], while in Morocco, it was 55.8% [22]. Therefore, an increasing trend in the use of smartphones was noticed. Differences in the prevalence of smartphone addiction between students in different countries could be due to different social and cultural surroundings.

The present study had several limitations. Firstly, it was conducted on a convenience sample of medical students, and the results of the study cannot be generalized to students in different fields. Future studies should be conducted among students in different fields and in the Serbian population to provide specific information regarding smartphone addiction. Secondly, the cut-off scores used for the Serbian version of the SAS-SV were based on the original scale. Although recommended cut-off values were widely used by researchers in different study populations (from children to adults), it is highly recommended to assess the predictive validity of this scale and report adequate cut-off scores in males and females. Thirdly, indicators of smartphone use were assessed through self-reporting, not objectively recorded data. It is recommended that future studies include objectively recorded data on the use of smartphones (e.g., acquired via a smartphone application), since the use of self-report questionnaires could lead to underestimation or overestimation of participants’ use of smartphones. However, the SAS-SV is among the most widely used and translated instruments to assess smartphone addiction. Widespread use of the SAS-SV could provide a unified approach to data collection, as well as its comparability.

## 5. Conclusions

The Serbian version of the SAS-SV is a reliable and valid instrument for detecting potential smartphone addiction. Several hours spent on a smartphone and a prevalence of addiction of 19.5% among medical students suggested that there is a need for further research on excessive smartphone usage and its drivers and consequences.

## Figures and Tables

**Table 1:** Item analysis and internal consistency of the SAS-SV in Serbian (*n* = 323 students).

Original Statement	M	SD	Corrected Item—Total Correlation	Cronbach’s Alpha if Item Deleted
Q1. Missing planned work due to smartphone use	2.56	1.53	0.69	0.88
Q2. Having a hard time concentrating in class, while doing assignments, or while working due to smartphone use *	2.22	1.35	0.65	0.88
Q3. Feeling pain in the wrists or at the back of the neck while using a smartphone *	1.70	1.13	0.52	0.89
Q4. Won’t be able to stand not having a smartphone *	2.55	1.68	0.65	0.88
Q5. Feeling impatient and fretful when I am not holding my smartphone *	2.26	1.44	0.70	0.88
Q6. Having my smartphone in my mind even when I am not using it *	1.84	1.20	0.73	0.88
Q7. I will never give up using my smartphone even when my daily life is already greatly affected by it *	2.35	1.44	0.59	0.88
Q8. Constantly checking my smartphone so as not to miss conversations between other people on Twitter or Facebook *	2.21	1.38	0.59	0.88
Q9. Using my smartphone longer than I had intended *	3.57	1.65	0.64	0.88
Q10. The people around me tell me that I use my smartphone too much *	2.26	1.43	0.64	0.88

M, mean; SD, standard deviation. * Min = 1. max = 6.

**Table 2:** Principal component analysis of SAS-SV in Serbian (*n* = 323 students).

		Rotated Component Matrix ^a^		Component Matrix ^b^
Question		Component		Component
	Extraction	1	2	1
Q1	0.803	0.181	0.878	0.748
Q2	0.724	0.201	0.826	0.727
Q3	0.448	0.228	0.630	0.606
Q4	0.734	0.834	0.196	0.728
Q5	0.728	0.802	0.291	0.773
Q6	0.662	0.651	0.487	0.805
Q7	0.620	0.768	0.171	0.665
Q8	0.451	0.535	0.406	0.666
Q9	0.534	0.403	0.609	0.716
Q10	0.536	0.440	0.585	0.725

Extraction method: principal component analysis. ^a^ Two components extracted; rotation method: Varimax with Kaiser normalization. ^b^ One component extracted (fixed).

**Table 3:** Exploratory factor analysis of SAS-SV in Serbian (*n* = 323 students).

Component	Initial Eigenvalues		
	Total	% of Variance	Cumulative%
1	5.154	51.538	51.538
2	1.085	10.853	62.391
3	0.769	7.688	70.079
4	0.673	6.734	76.813
5	0.567	5.674	82.486
6	0.454	4.540	87.026
7	0.432	4.320	91.346
8	0.372	3.716	95.062
9	0.263	2.634	97.696
10	0.230	2.304	100.000

Extraction method: principal component analysis.

**Table 4:** Correlation between the total SAS-SV score and time spent on smartphones (*n* = 323 students).

Average Time Spent on Smartphone	Hours M (SD)	*ρ* *	*p*
Smartphone usage on working days	3.83 (3.16)	0.31	<0.001
Smartphone usage on weekends	4.53(3.48)	0.32	<0.001
Social networks (working days)	2.69 (2.70)	0.39	<0.001
Social networks (weekends)	3.19 (2.98)	0.42	<0.001

M, mean; SD, standard deviation. * Spearman correlation coefficient; *p* value for Spearman correlation.

**Table 5:** Time spent on smartphones and social networks among “not addicted” and “addicted” students of medicine (*n* = 323 students).

Time Spent on Smartphone	Smartphone Addiction Status		*p*-Value *
	“Not Addicted” (*n* = 260) No. (%)	“Addicted” (*n* = 63) No. (%)	
Smartphone usage > 3 h (working days)	86 (33.6)	40 (63.5)	<0.001
Smartphone usage > 3 h (weekends)	102 (39.8)	48 (76.2)	<0.001
Social networks > 3 h (working days)	36 (15.2)	28 (46.7)	<0.001
Social networks > 3 h (weekends)	52 (21.7)	38 (61.3)	<0.001

* *p* value for χ^2^ test.

**Table 6:** Prevalence of smartphone addiction among students of medicine (*n* = 323 students).

	Smartphone Addiction Status		
	“Not Addicted” No. (%)	“Addicted” No. (%)	Total No. (%)
Male	86 (86.0)	14 (14.0)	100 (100)
Female	174 (78.0)	49 (22.0)	223 (100)
Total	260 (80.5)	63 (19.5)	323 (100)

Note: *p* value for χ^2^ test was 0.095.

## Data Availability

The data presented in this study are available upon request from the corresponding author. The data are not publicly available, since they are part of a larger dataset of a doctoral thesis that is in preparation.

## Supplementary Materials

The following supporting information can be download at: https://www.mdpi.com/article/10.3390/ijerph19031245/s1, Supplementary File S1: SAS-SV Serbian, Supplementary File S2: Sociodemographic characteristics and smartphone usage questionnaire.

## References

[1] Number of Smartphone Users Worldwide 2014–2020|Statista. https://www.statista.com/statistics/330695/number-of-smartphone-users-worldwide/.

[2] Cheever N.A., Rosen L.D., Carrier L.M., Chavez A. (2014). Out of Sight Is Not out of Mind: The Impact of Restricting Wireless Mobile Device Use on Anxiety Levels among Low, Moderate and High Users. Comput. Hum. Behav..

[3] Clayton R.B., Leshner G., Almond A. (2015). The Extended ISelf: The Impact of IPhone Separation on Cognition, Emotion, and Physiology. J. Comput.-Mediat. Commun..

[4] Demirci K., Akgönül M., Akpinar A. (2015). Relationship of Smartphone Use Severity with Sleep Quality, Depression, and Anxiety in University Students. J. Behav. Addict..

[5] Kim J.-H., Seo M., David P. (2015). Alleviating Depression Only to Become Problematic Mobile Phone Users: Can Face-to-Face Communication Be the Antidote?. Comput. Hum. Behav..

[6] Kwon M., Lee J.-Y., Won W.-Y., Park J.-W., Min J.-A., Hahn C., Gu X., Choi J.-H., Kim D.-J. (2013). Development and Validation of a Smartphone Addiction Scale (SAS). PLoS ONE.

[7] (2013). Diagnostic and Statistical Manual of Mental Disorders: DSM-5.

[8] ICD-11. https://icd.who.int/en.

[9] Griffiths M. (2005). A ‘Components’ Model of Addiction within a Biopsychosocial Framework. J. Subst. Use.

[10] Griffiths M. (2000). Does Internet and Computer “Addiction” Exist? Some Case Study Evidence. CyberPsychol. Behav..

[11] Harris B., Regan T., Schueler J., Fields S.A. (2020). Problematic Mobile Phone and Smartphone Use Scales: A Systematic Review. Front. Psychol..

[12] Lin Y.-H., Chang L.-R., Lee Y.-H., Tseng H.-W., Kuo T.B.J., Chen S.-H. (2014). Development and Validation of the Smartphone Addiction Inventory (SPAI). PLoS ONE.

[13] Merlo L.J., Stone A.M., Bibbey A. (2013). Measuring Problematic Mobile Phone Use: Development and Preliminary Psychometric Properties of the PUMP Scale. J. Addict..

[14] Kuss D., Harkin L., Kanjo E., Billieux J. (2018). Problematic Smartphone Use: Investigating Contemporary Experiences Using a Convergent Design. Int. J. Environ. Res. Public Health.

[15] Rosen L.D., Whaling K., Carrier L.M., Cheever N.A., Rokkum J. (2013). The Media and Technology Usage and Attitudes Scale: An Empirical Investigation. Comput. Hum. Behav..

[16] Bock B.C., Lantini R., Thind H., Walaska K., Rosen R.K., Fava J.L., Barnett N.P., Scott-Sheldon L.A. (2016). The Mobile Phone Affinity Scale: Enhancement and Refinement. JMIR Mhealth Uhealth.

[17] Kwon M., Kim D.-J., Cho H., Yang S. (2013). The Smartphone Addiction Scale: Development and Validation of a Short Version for Adolescents. PLoS ONE.

[18] Mescollotto F.F., de Castro E.M., Pelai E.B., Pertille A., Bigaton D.R. (2019). Translation of the Short Version of the Smartphone Addiction Scale into Brazilian Portuguese: Cross-Cultural Adaptation and Testing of Measurement Properties. Braz. J. Phys. Ther..

[19] De Pasquale C., Sciacca F., Hichy Z. (2017). Italian Validation of Smartphone Addiction Scale Short Version for Adolescents and Young Adults (SAS-SV). PSYCH.

[20] Akın A., Altundağ Y., Turan M.E., Akın U. (2014). The Validity and Reliability of the Turkish Version of the Smart Phone Addiction Scale-Short Form for Adolescent. Procedia-Soc. Behav. Sci..

[21] Lopez-Fernandez O. (2017). Short Version of the Smartphone Addiction Scale Adapted to Spanish and French: Towards a Cross-Cultural Research in Problematic Mobile Phone Use. Addict. Behav..

[22] Sfendla A., Laita M., Nejjar B., Souirti Z., Touhami A.A.O., Senhaji M. (2018). Reliability of the Arabic Smartphone Addiction Scale and Smartphone Addiction Scale-Short Version in Two Different Moroccan Samples. Cyberpsychol. Behav. Soc. Netw..

[23] Beaton D.E., Bombardier C., Guillemin F., Ferraz M.B. (2000). Guidelines for the Process of Cross-Cultural Adaptation of Self-Report Measures. Spine.

[24] Chen B., Liu F., Ding S., Ying X., Wang L., Wen Y. (2017). Gender Differences in Factors Associated with Smartphone Addiction: A Cross-Sectional Study among Medical College Students. BMC Psychiatry.

[25] Field A.P. (2009). Discovering Statistics Using SPSS: And Sex, Drugs and Rock “n” Roll.

[26] Terwee C.B., Bot S.D.M., de Boer M.R., van der Windt D.A.W.M., Knol D.L., Dekker J., Bouter L.M., de Vet H.C.W. (2007). Quality Criteria Were Proposed for Measurement Properties of Health Status Questionnaires. J. Clin. Epidemiol..

[27] Haug S., Castro R.P., Kwon M., Filler A., Kowatsch T., Schaub M.P. (2015). Smartphone Use and Smartphone Addiction among Young People in Switzerland. J. Behav. Addict..

[28] Luk T.T., Wang M.P., Shen C., Wan A., Chau P.H., Oliffe J., Viswanath K., Chan S.S., Lam T.H. (2018). Short Version of the Smartphone Addiction Scale in Chinese Adults: Psychometric Properties, Sociodemographic, and Health Behavioral Correlates. J. Behav. Addict..

[29] Lepp A., Barkley J.E., Karpinski A.C. (2015). The Relationship Between Cell Phone Use and Academic Performance in a Sample of U.S. College Students. SAGE Open.

[30] King A.L.S., Pádua M.K., Gonçalves L.L., Santana de Souza Martins A., Nardi A.E. (2020). Smartphone Use by Health Professionals: A Review. Digit. Health.

[31] Lin Y.-H., Pan Y.-C., Lin S.-H., Chen S.-H. (2017). Development of Short-Form and Screening Cutoff Point of the Smartphone Addiction Inventory (SPAI-SF). Int. J. Methods Psychiatr. Res..

[32] Lin Y.-H., Chiang C.-L., Lin P.-H., Chang L.-R., Ko C.-H., Lee Y.-H., Lin S.-H. (2016). Proposed Diagnostic Criteria for Smartphone Addiction. PLoS ONE.

[33] Horvath J., Mundinger C., Schmitgen M.M., Wolf N.D., Sambataro F., Hirjak D., Kubera K.M., Koenig J., Christian Wolf R. (2020). Structural and Functional Correlates of Smartphone Addiction. Addict. Behav..

[34] Panova T., Carbonell X. (2018). Is Smartphone Addiction Really an Addiction?. J. Behav. Addict..

[35] Billieux J., Maurage P., Lopez-Fernandez O., Kuss D.J., Griffiths M.D. (2015). Can Disordered Mobile Phone Use Be Considered a Behavioral Addiction? An Update on Current Evidence and a Comprehensive Model for Future Research. Curr. Addict. Rep..

[36] Billieux J., Schimmenti A., Khazaal Y., Maurage P., Heeren A. (2015). Are We Overpathologizing Everyday Life? A Tenable Blueprint for Behavioral Addiction Research. J. Behav. Addict..

[37] Venkatesh E., Jemal M.Y.A., Samani A.S.A. (2019). Smart Phone Usage and Addiction among Dental Students in Saudi Arabia: A Cross Sectional Study. Int. J. Adolesc. Med. Health.

