# Supplementary material for: The Validity and Reliability of the Serbian Version of the Smartphone Addiction Scale—Short Version
Source: Int J Environ Res Public Health. 2022 Jan 22;19(3):1245. doi: 10.3390/ijerph19031245 (PMC8835088; doi:10.3390/ijerph19031245)
Supplement: Supplementary file 1 [file ijerph-19-01245-s001.zip › ijerph-1530722-supplementary.pdf]

Supplementary File S1

Serbian Version of SAS-SV

	**Stavke**	**Ni malo se ne slažem**	**Ne slažem se**	**Delimično se ne slažem**	**Više se slažem nego što se ne slažem**	**Slažem se**	**U potpunosti se slažem**
1	Ne ispunjavam planirani posao zbog korišćenja pametnog telefona	1	2	3	4	5	6
2	Teško se koncentrišem na času, dok radim zadatke, ili na poslu zbog korišćenja pametnog telefona	1	2	3	4	5	6
3	Osećam bol u ručnom zglobu ili u zadnjem delu vrata dok koristim pametni telefon	1	2	3	4	5	6
4	Ne mogu da živim bez pametnog telefona	1	2	3	4	5	6
5	Osećam se nelagodno i nesrećno kad nemam svoj pametni telefon pri ruci	1	2	3	4	5	6
6	Razmišljam o svom pametnom telefonu čak i kad ga ne koristim	1	2	3	4	5	6
7	Nikada ne bih odustao/la od korišćenja pametnog telefona čak i kad u velikoj meri utiče na moj svakodnevni život	1	2	3	4	5	6
8	Konstantno proveravam svoj telefon da ne bih propustio/la konverzaciju između drugih ljudi na Fejsbuku, Tviteru…	1	2	3	4	5	6
9	Koristim pametni telefon duže nego što planiram	1	2	3	4	5	6
10	Ljudi iz mog okruženja mi govore da previše koristim svoj pametni telefon	1	2	3	4	5	6

Supplementary File S2

I Demographic data

1. Sex 1. Male 2. Female

2. Date of birth (day, month, year) ___________________________

3. Place of permanent residence _____________________Municipality________________________

4. Place of residence during the last 30 days during the study

   1. With parents 4. Rented flat/room

   2. In their own apartment 5. With friends/cousins

   3. Student's dorm 6. Other, state where______________________

6. What year are you in faculty? First Second Third Fourth Fifth Sixth

7. Grade point average: __________

8. How would you describe your socio-economic situation? a) good b) average c) bad

II Smartphone usage patterns

1. How many hours daily do you spend using a smartphone? (working day) __________

2. How many hours daily do you spend using social networks on smartphone? (working day) _____

3. How many hours daily do you spend using a smartphone? (weekend) ___________

4. How many hours daily do you spend using social networks on smartphone? (weekend) _______
